# Antioxidant Mechanism of *Lactiplantibacillus plantarum* KM1 Under H_2_O_2_ Stress by Proteomics Analysis

**DOI:** 10.3389/fmicb.2022.897387

**Published:** 2022-06-27

**Authors:** Yuan Tian, Yu Wang, Nan Zhang, Minmin Xiao, Jing Zhang, Xinyue Xing, Yue Zhang, Yuling Fan, Xia Li, Bo Nan, Yuhua Wang, Jingsheng Liu

**Affiliations:** ^1^College of Food Science, Jilin Agricultural University, Changchun, China; ^2^Jilin Province Innovation Center for Food Biological Manufacture, Jilin Agricultural University, Changchun, China; ^3^National Processing Laboratory for Soybean Industry and Technology, Changchun, China; ^4^National Engineering Laboratory for Wheat and Corn Deep Processing, Changchun, China

**Keywords:** *Lactiplantibacillus plantarum*, antioxidant, proteomics, oxidative stress, probiotic

## Abstract

*Lactiplantibacillus plantarum* KM1 was screened from natural fermented products, which had probiotic properties and antioxidant function. The survival rate of *L. plantarum* KM1 was 78.26% at 5 mM H_2_O_2_. In this study, the antioxidant mechanism of *L. plantarum* KM1 was deeply analyzed by using the proteomics method. The results demonstrated that a total of 112 differentially expressed proteins (DEPs) were screened, of which, 31 DEPs were upregulated and 81 were downregulated. The Kyoto Encyclopedia of Genes and Genomes (KEGG) enrichment analysis indicated that DEPs participated in various metabolic pathways such as pyruvate metabolism, carbon metabolism, trichloroacetic acid cycle, amino acid metabolism, and microbial metabolism in diverse environments. These metabolic pathways were related to oxidative stress caused by H_2_O_2_ in *L. plantarum* KM1. Therefore, the antioxidant mechanism of *L. plantarum* KM1 under H_2_O_2_ stress provided a theoretical basis for its use as a potential natural antioxidant.

## Introduction

Oxidative stress is an imbalance between the production of reactive oxygen species (ROS) and the antioxidant defense of cells (Jacob et al., 2013). Oxidative stress plays an important role in human health, which is related to many diseases (Preiser, 2012), such as chronic diseases, cardiovascular diseases, atherogenesis, and neurodegenerative diseases (Zhou et al., 2008; Sharifpanah and Sauer, 2016). Antioxidants improve oxidative stress as a target to treat these diseases, which are divided into natural antioxidants and synthetic antioxidants according to their sources. However, most of synthetic antioxidants were proved to have side effects (Kulawik et al., 2013). Therefore, it is necessary to find safe and reliable natural antioxidants. Studies showed that lactic acid bacteria (LAB) have antioxidant effects (Lin and Yen, 1999; Wang et al., 2012, 2021; Teng et al., 2020).

Lactic acid bacteria are generally recognized as safe (GRAS) microorganisms (Azat et al., 2016) and also important food resources with certain probiotic activities, including regulating gastrointestinal, lowering cholesterol, alleviating diseases, and relieving lactose intolerance and anti-oxidation (Park et al., 2002; Mishra et al., 2015). As an inexpensive natural antioxidant, *Lactiplantibacillus plantarum* can scavenge free radicals and exert its antioxidant functions to regulate oxidative stress (Teng et al., 2020). Additionally, the antioxidant properties of *L. plantarum* were also attributed to the intracellular antioxidant enzyme system (Liu et al., 2021). Under oxidative stress, antioxidant enzymes such as catalase, NADH peroxidase, and thioredoxin reductase were activated to resist oxidative stimulation. *L. plantarum* MA2 with the antioxidant capacity *in vitro* was identified, and the antioxidant-related genes including cat, gshR, and npx were upregulated under hydrogen peroxide (H_2_O_2_) challenge (Tang et al., 2017). The gshR and npx were upregulated in *L. plantarum* AR113 with probiotic activities after H_2_O_2_ treatment (Lin et al., 2020). In addition, many genes in various pathways changed from the global transcriptomics perspective of *L. plantarum* CAUH2 in response to H_2_O_2_ (Zhai et al., 2020). It is a particularly important method to use H_2_O_2_ as an oxidant to screen LAB with antioxidant function. In this way, LAB can exert its antioxidant function and use its antioxidant mechanism to alleviate the negative effects of oxidative stress. However, the antioxidant mechanism of LAB is not yet clear, and few studies have reported protein changes associated with oxidative stress.

Moreover, proteomics becomes a hot topic in current genomics research. Proteomics not only characterizes and identifies large-scale proteins but also determines protein activities, functions, and interactions (Vittaladevaram, 2021). More studies have carried out comparative proteomic analysis based on stress conditions. To study the changes and functional roles of the differentially expressed proteins (DEPs), the DEPs of bacteria were quantitatively analyzed through the TMT labeling quantitative proteomics technology (O'Connor et al., 2000). The potential mechanism of nitrite degradation by *Lactobacillus fermentum* RC4 under nitrite stress was analyzed using proteomics (Zeng et al., 2019). Proteomics technology was used to analyze the DEP changes of *Phaffia rhodozyma* under TiO_2_ stress and to clarify the metabolic mechanism of the strain promoting astaxanthin synthesis (Zhang et al., 2020). When faced with various stress conditions, strains effectively cope with unfavorable conditions under the action of DEPs. Therefore, the mechanism of strains under oxidative stress can be more clearly reflected based on the proteomic analysis.

*L. plantarum* KM1 isolated from natural fermented food tolerated high concentrations of H_2_O_2_ and satisfied the basic criteria for probiotic screening. The antioxidant ability of this strain was evaluated, and its antioxidant mechanism was explored from the proteomics perspective, which provided a theoretical foundation for its use as a natural antioxidant for functional foods.

## Materials and Methods

### Strain Isolation and Identification

A piece of naturally fermented food was directly taken into sterilized Mann, Rogosa and Sharpe (MRS) medium (Qingdao Hi-Tech Park Haibo Biotechnology Co., Ltd., China) and cultivated at 37°C for 24 h. The strains were inoculated at 3% inoculum into MRS medium containing 10 mM H_2_O_2_ for preliminary screening. At this time, the surviving strains were inoculated into a new MRS liquid medium for 24 h, which were the primary screening strains. The strains were repeatedly isolated and purified on MRS agar plates and cultured at 37°C for 48 h. By observing the morphology of the colony and using a microscope to observe, a single colony was separated and purified to obtain a pure strain. The purified strains were subjected to bacterial 16S-rDNA identification and sequencing. The genomic DNA of the strain was extracted and then performed PCR amplification with universal primers. Finally, the PCR product was recovered and sequenced. The sequencing results came from the Shanghai Biotechnology Engineering Co., Ltd. Sequencing results were compared on the NCBI website for homology comparison, and a phylogenetic tree was constructed.

### Determination of H_2_O_2_ Concentration

A pure culture of KM1 was inoculated into the MRS broth contained with different concentrations of H_2_O_2_ (i.e., 1.25, 2.5, 5.0, and 10.0 mM). The same amount of physiological saline was added to the MRS broth as the control group. KM1 was cultured at 37°C in incubators (Shanghai Zhicheng Analytical Instrument Manufacturing Co., Ltd.). The samples were shaken briefly, and then the OD value was measured at 600 nm every 2 h.

### Determination of Resistance to Acid and Bile Salts

The determination of acid resistance and bile salt resistance was slightly modified according to the reported method (Ribeiro et al., 2018). *L. plantarum* KM1 was inoculated into the MRS medium (2%, w/w), in which the pH was adjusted to 4.0, 3.0, 2.5, and 2.0 with HCl. The bile salt resistance was determined by inoculating *L. plantarum* KM1 into the MRS medium containing 0.1 and 0.2% bile salt (Solarbio, China). The normal medium was used as the control group. The above cultures were then cultured at 37°C, and samples were taken at 0, 2, and 4 h, respectively, and spread on the MRS solid medium for 48 h to count the number of colonies. Each experiment was performed in triplicate.

### Antibiotic Sensitivity

According to previous reports, the micro broth dilution method was used to determine the antibiotic sensitivity of strains (Wang et al., 2021). Five antibiotics were accurately weighed 5,120 μg, including kanamycin sulfate, streptomycin sulfate, tetracycline HCl, chloramphenicol, and ampicillin trihydrate (Solarbio, China). The above five antibiotics were serially diluted in the range of 256–1 μg/ml, and the final concentration of the antibiotic solution was 256, 128, 64, 32, 16, 8, 4, 2, and 1 μg/ml. Then, 2% bacterial solution was added to the diluted solution. Among them, the blank group without antibiotics and bacterial solution and the control group without antibiotics were set up. The cultures were incubated at 37°C for 24 h to determine its minimum inhibitory concentration (MIC). Each experiment was conducted in triplicate.

### Antioxidant Activity

#### ABTS Radical Scavenging

The determination method of ABTS free radicals was modified according to the previous method (Thaipong et al., 2006). ABTS free radical ion working solution was a mixture of 7.4 mM ABTS (Shanghai McLean Biochemical Technology Co., Ltd., China) stock solution and 2.6 mM potassium persulfate solution and must be placed at room temperature in the dark condition for 12 h. The sample was mixed with the diluted ABTS working solution and reacted at 37°C for 30 min, and an equal volume of 95% ethanol was used as a control group instead of the sample. The absorbance was measured at 734 nm and marked them as A_s_ and A_0_, respectively. Each experiment was conducted in triplicate. The ABTS radical scavenging rate was calculated with the following formula:


$$\begin{array}{l} ABTS\;radical\;scavenging\;rate\left(\%\right)=\frac{\left(A_{0}-A_{s}\right)}{A_{0}}\times 100\% \end{array}$$

#### Determination of Reducing Activity

The determination of the reducing activity was improved on the basis of the previous reported method (Li et al., 2008). The sample (0.5 ml) was added to 1.5 ml of phosphate buffer (0.2 M, Shanghai Corning Management Co., Ltd., China) and 1.5 ml of 1% (w/v) potassium ferricyanide (Beijing chemical factory, China). The mixture was placed in a water bath at 50°C for 20 min and quickly cooled to room temperature. The mixture was added to 1.5 ml of trichloroacetic acid (TCA, 10%, Tianjin Guangfu Fine Chemical Research Institute, China) and after shaking well, centrifuged at 8,000 rpm for 10 min to get the supernatant. The supernatant was mixed with the same amount of physiological saline, and 1 ml of ferric chloride solution was added. After standing for 10 min, the absorbance values were read at 700 nm. Different concentrations of L-ascorbic acid (Beijing Dingguo Biotechnology Co., Ltd., China) were made into a standard curve. Each experiment was conducted in triplicate.

#### Hydroxyl Radical Scavenging

Hydroxyl radical scavenging was conducted according to the Nanjing Jiancheng kit instructions.

#### DPPH Radical Scavenging Activity Assay

DPPH radical scavenging activity was carried out using the Wei Tang's method (Luan et al., 2021) and modified. The prepared sample was mixed thoroughly at 1:1 with DPPH solution formulated with absolute ethanol. The mixed sample was reacted for 30 min at room temperature under dark conditions and centrifuged at 6,000 rpm for 10 min, and the supernatant was taken to measure the absorbance values (A_s_) at 517 nm. At the same time, an equal volume of absolute ethanol replaced the DPPH solution to measure the absorbance value and recorded it as A_b_. The control group replaced the sample with an equal of distilled water volume, and the measured absorbance value was A_c_. Each experiment was conducted in triplicate. The DPPH radical scavenging rate was calculated with the following formula:


$$\begin{array}{l} DPPH\;radical\;scavenging\;rate\left(\%\right)=\left[1-\frac{\left(A_{s}-A_{b}\right)}{A_{c}}\right]\times 100\% \end{array}$$

### Proteomic Analysis of *L. plantarum* KM1 Under H_2_O_2_ Stress

#### Protein Extraction and Peptide Digestion

After 18 h of incubation, the sample group (5 mM H_2_O_2_) and control group (0 mM H_2_O_2_) were obtained, respectively. Each group contained 3 biological replicates for a total of 6 samples. Control 1, Control 2, and Control 3 were labeled with 126, 127, or 128 TMT reagents, respectively. H_2_O_2_ 1, H_2_O_2_ 2, and H_2_O_2_ 3 were labeled with 129, 130, and 131 TMT reagents, respectively. The protein was extracted by SDT (4% sodium lauryl sulfate (SDS), 100 mM Tris/HCl pH 7.6, 0.1M DTT) lysis method (Wiśniewski et al., 2009), and then total protein was quantified using the BCA protein assay kit (Thermo Scientific, USA). Protein was completely dissolved in SDS according to the filter-aided proteome preparation (FASP) method (Wiśniewski et al., 2009) for trypsin digestion. The peptides were desalted by C18 Cartridge after enzymatic hydrolysis and then freeze-dried and reconstituted with adding 40 μl dissolution buffer. The peptides were quantified at the absorbance of OD_280_.

#### TMT Labeling and Strong Cation Exchange Chromatographic Classification

Furthermore, 100 μg peptides of each sample were labeled according to the instructions of the TMT labeling kit (Thermo Fisher, USA). The labeled peptides from each group were pooled and purified using an AKTA Purifier 100 (GE Healthcare, USA). The sample was loaded to the column equilibrated with buffer A (10 mM KH_2_PO_4_, 25% ACN, pH 3.0) and B (10 mM KH_2_PO_4_, 500 mM KCl, 25% ACN, pH 3.0). The chromatographic column (5 μm, 200 Å, PolyLCInc, Maryland, U.S.A.) was equilibrated with liquid A, and the sample was loaded from the injector to the chromatographic column for separation at a flow rate of 1 ml/min. The liquid-phase gradient was as follows: 0–25 min, the linear gradient of liquid B was from 0 to 10%; 25–32 min, the linear gradient of liquid B was from 10 to 20%; 32–42 min, the linear gradient of liquid B was from 20 to 45%; 42–47 min, the linear gradient of B solution was from 45 to 100%; 47–52 min, 52–60 min B solution was maintained at 100%; after 60 min, B solution was reset to 0%. During the elution process, the absorbance value at 214 nm was monitored, and the elution fractions were collected every 1 min, respectively, lyophilized and desalted using a C18 Cartridge.

#### Liquid Chromatography With Tandem Mass Spectrometry Data Acquisition

Each sample fraction was separated by high-performance liquid chromatography (HPLC) liquid system Easy nLC (Thermo Scientific, USA) with a separation column (Thermo scientific EASY column, 75 μm × 10 cm, 3 μm, C18). After the sample was chromatographed, it was analyzed using the Q-Exactive mass spectrometer (Thermo, USA) for 60 min. The peptides were detected by the positive ion mode, the scanning range was set to 300–1,800 m/z, the resolution was 70,000 (at m/z 200), automatic gain control (AGC) target was 3e6, maximum IT was 10 ms, and the dynamic exclusion time was 40 s. The mass-to-charge ratios of peptides and peptide fragments were calculated by collecting 10 fragment maps (MS2 scan) after each full scan, and the MS resolution was 17,500 (at m/z 200).

#### Protein Identification and Quantitative Analysis

Mass spectrometry (MS) analysis was performed with software Mascot 2.2 and Proteome Discoverer 1.4 for library identification and quantitative analysis with MS/MS ion search. The search parameters for protein identification and relative quantification were as follows: The maximum number of missed cleavage sites allowed was 2. Fixed modifications were carbamidomethyl (C), TMT 10plex (N-term), TMT 10plex (K), and variable modifications were oxidation (M), TMT 10plex (Y). The peptide mass tolerance was ±20 ppm, and fragment mass tolerance was 0.1 Da.

The database mode used to calculate false discovery rate (FDR) was set to Decoy, and FDR ≤ 0.01 was used as the screening criterion for credible proteins. The ratios (H_2_O_2_/control) were calculated as the median of only unique peptides of the protein normalizes all peptide ratios by the median protein ratio. The median protein ratio should be 1 after the normalization. The relative expression of the DEP was the relative expression of the sample group relative to the internal reference protein, which was the ratio of the signal intensity value of the reporter ion peak of the sample labeling channel and the internal standard sample in the experimental design after correction.

#### Bioinformatics Analysis

The target protein set used the Omicsbean software for Gene Ontology (GO) function annotation and Kyoto Encyclopedia of Genes and Genomes (KEGG) signal pathway. GO and KEGG pathway enrichment analyses of the DEPs were performed by the Fisher's exact test method. The detailed information of GO function annotation was obtained by consulting the related database (http://www.geneontology.org/). Information about the KEGG pathway could be accessed through the KEGG database (http://www.kegg.jp/). The STRING database (http://string-db.org) was used to analyze the protein-protein interactions (PPIs) of *L. plantarum* KM1 including direct physical interactions and functional correlations.

### RT-qPCR Assay

Totally, 50 ml of the cultured bacterial solution was centrifuged at 5,000 *g* and transferred to a non-enzyme centrifuge tube, and 1 ml of Trizol was added and allowed to stand for 15 min after repeated pipetting and lysis. Furthermore, 200 μl of ice-cold chloroform was added to the above lysate and let stand on ice for 5 min. After centrifugation at 12,000 *g* for 15 min at 4°C, the upper aqueous phase was carefully aspirated, an equal volume of precooled isopropanol was added, and the mixture was evenly mixed and placed on ice for 10 min. After centrifugation at 12,000 *g* for 10 min at 4°C, the supernatant was carefully discarded, and 75% ethanol prepared with 1 ml of DEPC water was added to wash the white precipitate and let stand for 5 min. After centrifugation at 12,000 *g* for 5 min at 4°C, the supernatant was discarded, and the pellet was allowed to stand at room temperature for 5 min to dry the pellet. After adding 20 μl of DEPC water to dissolve the precipitate, the extracted RNA is reverse-transcribed into cDNA according to the instructions of the cDNA kit. According to the instructions of TB Green™Premix Ex Taq™II kit (Japan Takara Biological Co., Ltd.), samples were added and mixed well for on-machine test. The amplification conditions are shown in Supplementary Table S1. 16sRNA was used as the internal reference, and the primer design was shown in Supplementary Table S2.

### Statistical Analysis

The experimental data were expressed as the mean ± SEM. The significance analysis of experimental data was used *t*-test and one-way ANOVA (and non-parametric or mixed) by Prism 8.0. The PPI network of DEPs was analyzed using the STRING software 11.0.

## Results

### Screening and Identification of Hydrogen Peroxide-Tolerant Strains

**Figure 1:**
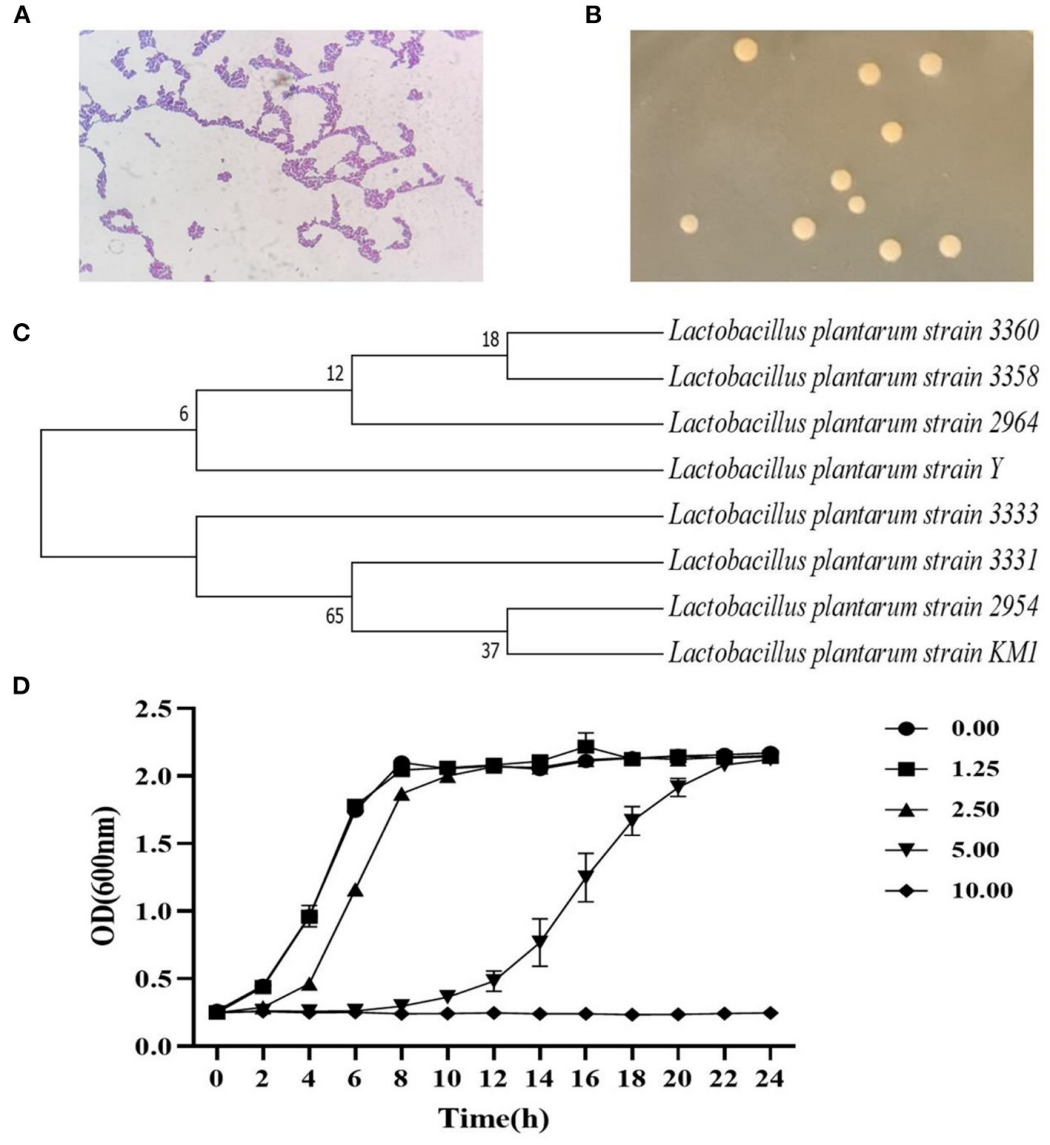
The identification of strain by the 16S rDNA sequence and its growth curve. The strain characteristics (100×) **(A)**, colony morphology **(B)**, and the phylogenetic tree **(C)** of *L. plantarum* KM1. Growth of *L. plantarum* KM1 under the stress of different concentrations of H_2_O_2_ **(D)**.

The strain was separated and purified from natural fermented yogurt, and morphology was observed under a microscope (Figure 1A). The colony of this strain was round, smooth, dense, and white (Figure 1B). The strain was identified using 16S-rDNA sequencing, and a phylogenetic tree was constructed based on the sequence (Figure 1C). The results showed that this strain had high system homology (99%) with *L. plantarum* named *L. plantarum* KM1.

The growth of *L. plantarum* KM1 were determined at 1.25–10.00 mM H_2_O_2_ in MRS medium. The growth of KM1 was gradually inhibited with increasing the H_2_O_2_ concentration, and it grew well under 1.25–5.00 mM H_2_O_2_, while it was completely inhibited at 10.00 mM (Figure 1D). Therefore, *L. plantarum* KM1 can tolerate a maximum concentration of 5.00 mM H_2_O_2_.

### Acid and Bile Salt Resistance Properties of *L. plantarum* KM1

**Figure 2:**
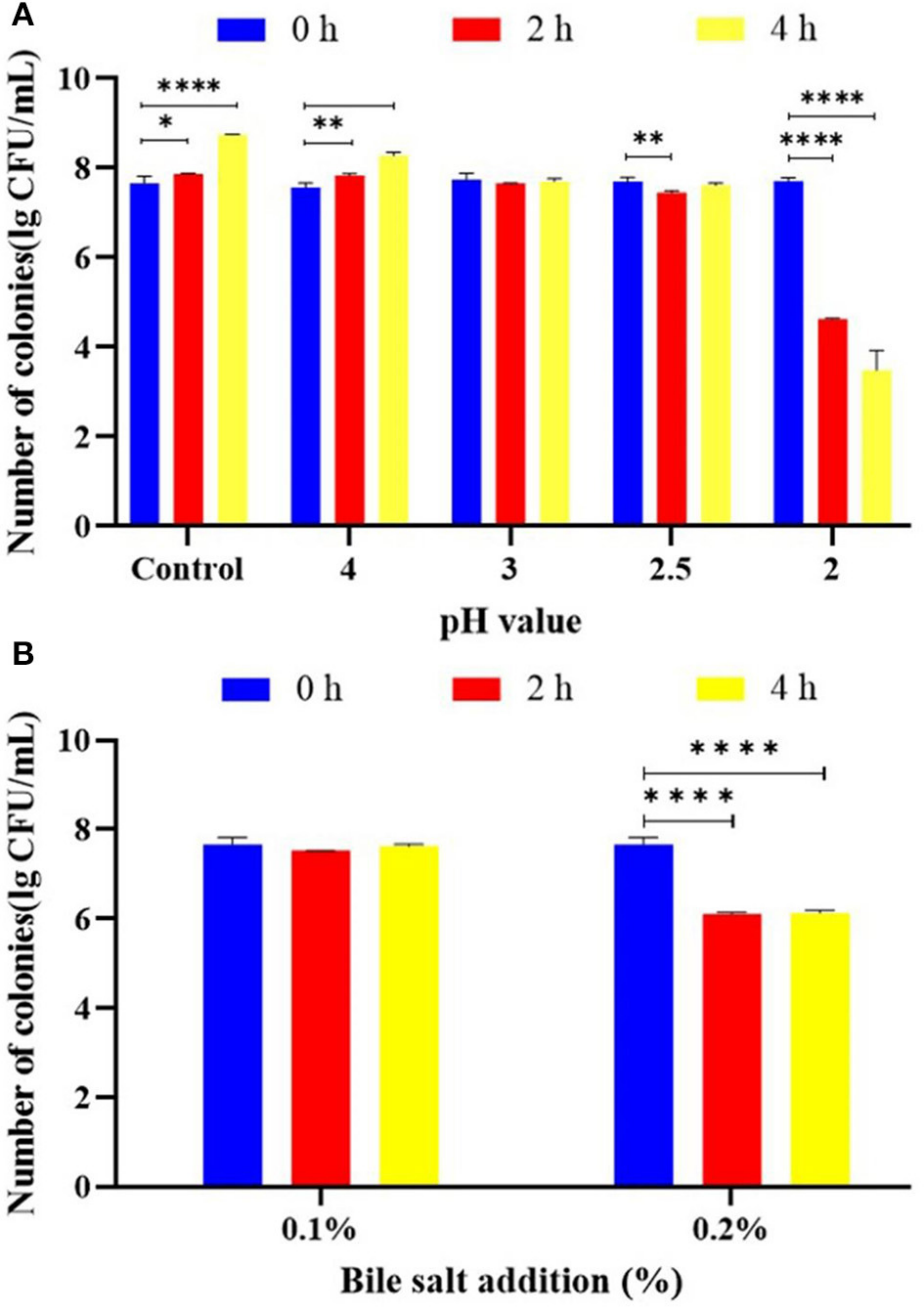
Probiotic properties of *L. plantarum* KM1. The acid tolerance **(A)** and bile salt tolerance **(B)** of *L. plantarum* KM1. *p < 0.05, **p < 0.01, and ****p < 0.0001.

It is an important probiotic activity to adapt to the human gastric acid and the bile salt environment after ingestion. The acid resistance of *L. plantarum* KM1 was tested (Figure 2A). *L. plantarum* KM1 grew well under the condition of pH 2.5 or higher. Under the condition of pH 2.0, the number of viable colonies of the strain dropped significantly from 7.715 ± 0.041 lg CFU/ml to 4.626 ± 0.009 lg CFU/ml in 2 h and significantly reduced to 3.486 ± 0.257 lg CFU/ml in 4 h. *L. plantarum* KM1 was cultured in the MRS medium at 0.1 and 0.2% bile salt for 2 and 4 h (Figure 2B). The number of colonies of *L. plantarum* KM1 was 7.528 ± 0.003 lg CFU/ml after 2 h when bile salt was 0.1%, and increased to 7.629 ± 0.024 lg CFU/ml after 4 h. When the bile salt concentration was 0.2%, the number of *L. plantarum* KM1 decreased significantly from 7.664 ± 0.009 lg CFU/ml to 6.113 ± 0.019 lg CFU/ml in 2 h and 6.132 ± 0.037 lg CFU/ml in 4 h (*p* < 0.0001).

### Antibiotic Sensitivity of *L. plantarum* KM1

The MIC of *L. plantarum* KM1 against 5 antibiotics was measured (Table 1). *L. plantarum* KM1 was sensitive to five antibiotics, namely, kanamycin sulfate, streptomycin sulfate, tetracycline HCl, chloramphenicol, and ampicillin trihydrate. The MIC of *L. plantarum* KM1 against kanamycin sulfate and streptomycin sulfate was both >256 μg/ml. *L. plantarum* KM1 had an MIC of 8 μg/ml for chloramphenicol and 32 μg/ml for tetracycline HCl. *L. plantarum* KM1 had the smallest MIC value for ampicillin trihydrate (2 μg/ml).

**Table 1:** The minimum inhibitory concentration of *L. plantarum* KM1 to antibiotics.

**Strain**	**MIC (μg/mL)**				
	**Km**	**Sm**	**Cl**	**Te**	**Am**
*L. plantarum* KM1	>256	>256	8	32	2

*Km, Kanamycin Sulfate; Sm, Streptomycin Sulfate; Cl, Chloramphenicol; Te, Tetracycline HCL; Am, Ampicillin trihydrate*.

### Antioxidant Activity of *L. plantarum* KM1 in Different Component

**Figure 3:**
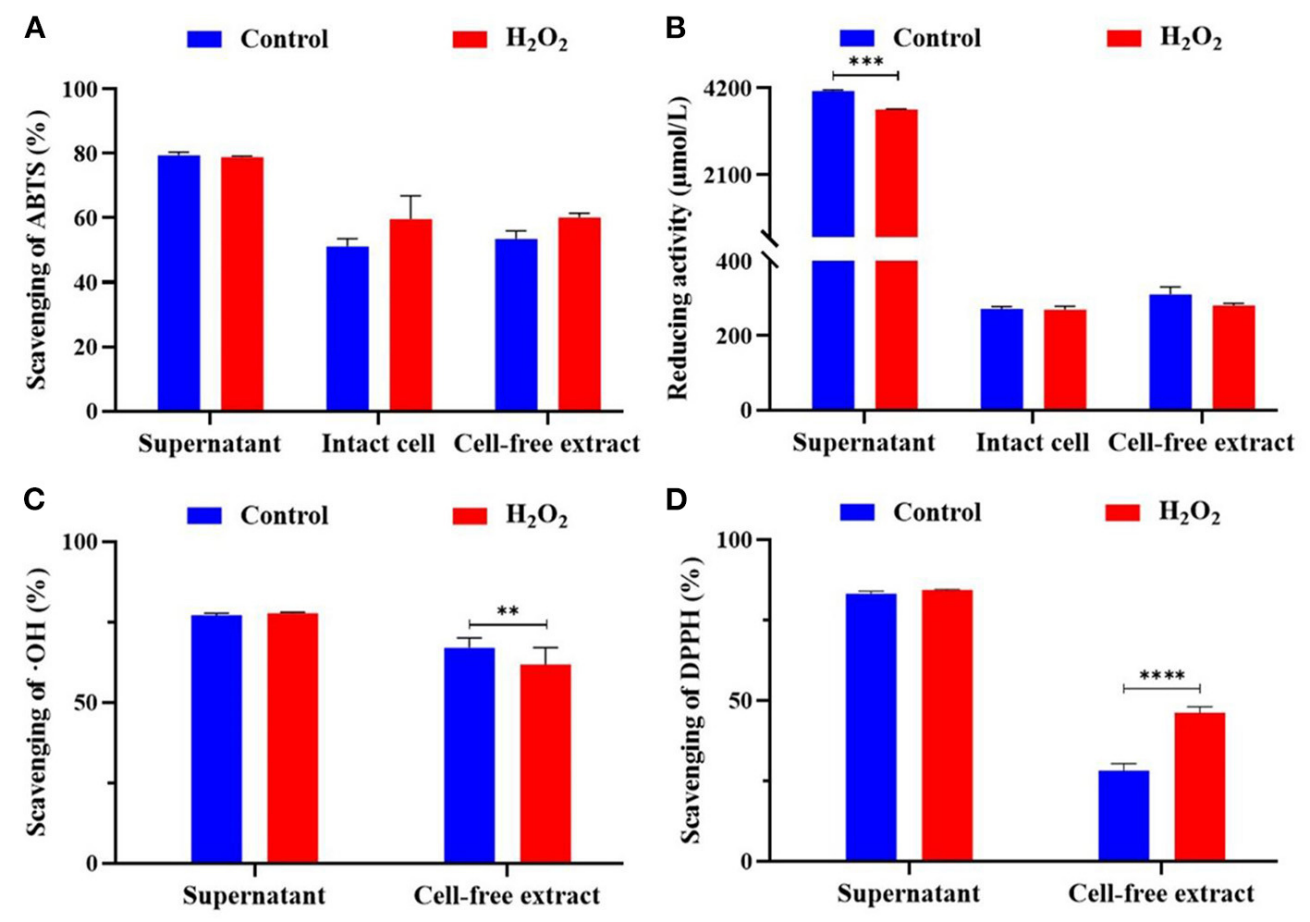
The antioxidant activity of *L. plantarum* KM1 different components. Scavenging of ABTS (%) **(A)**, reducing activity (μmol/L) **(B)**, scavenging of OH (%) **(C)**, and scavenging of DPPH (%) **(D)**. Free radical scavenging ability and reducing ability of fermentation supernatant minus the corresponding antioxidant evaluation of MRS medium itself. Control, the normal group; H_2_O_2_, hydrogen peroxide intervention group. **p < 0.01, ***p < 0.001, and ****p < 0.0001.

The scavenging rate of three free radicals (i.e., ABTS, ∙OH, and DPPH) and reducing activity of different components (i.e., supernatant, intact cell, and cell-free extract) with H_2_O_2_ were measured (Figure 3). The ABTS free radical scavenging rate of *L. plantarum* KM1 supernatant, intact cells, and cell-free extracts were 79.494 ± 0.984%, 51.129 ± 1.400%, and 53.489 ± 1.416%, respectively. There was no significant effect on the ABTS free radical scavenging rate of the three components of *L. plantarum* KM1 with 5 mM H_2_O_2_ treatment.

L-ascorbic acid was used as a standard curve to determine the standard curve of the reducing ability of *L. plantarum* KM1 (Figure 3B). The supernatant of *L. plantarum* KM1 had the strongest reducing activity (4,133.000 ± 23.334 μmol/L) of three components. The reducing activity of intact cells was 271.778 ± 4.006 μmol/L, and the cell-free extracts were 310.67 ± 12.019 μmol/L.

∙OH is the most active free radicals in ROS, which cause oxidative damage (Yang et al., 2017). The ∙OH scavenging rate of the KM1 supernatant was the highest (77.114 ± 0.595%) among two components (Figure 3C). The ∙OH scavenging rate of cell-free extract of *L. plantarum* KM1 was 66.963 ± 1.033%. After treatment with 5 mM H_2_O_2_, the ∙OH scavenging rate of the cell-free extract was significantly lower than the normal group (*p* < 0.01). *L. plantarum* KM1 could scavenge ∙OH and alleviate oxidative damage caused by ∙OH.

Based on the measurement of DPPH radicals, the scavenging rate of the supernatant of *L. plantarum* KM1 was 83.173 ± 0.775% (Figure 3D). The DPPH scavenging of the KM1 cell-free extract in control group was 28.173 ± 1.285%. The DPPH free radical scavenging rate of *L. plantarum* KM1 cell-free extract treated with 5 mM H_2_O_2_ was 46.240 ± 1.051%, which was significantly higher than that of the normal group.

### Proteomics Analysis of *L. plantarum* KM1 Under 5 mM H_2_O_2_

Proteomics analysis was performed to study the antioxidant mechanism of *L. plantarum* KM1 cultured in MRS with 5 mM H_2_O_2_ for 18 h.

#### Differentially Expressed Proteins Analysis of *L. plantarum* KM1

**Figure 4:**
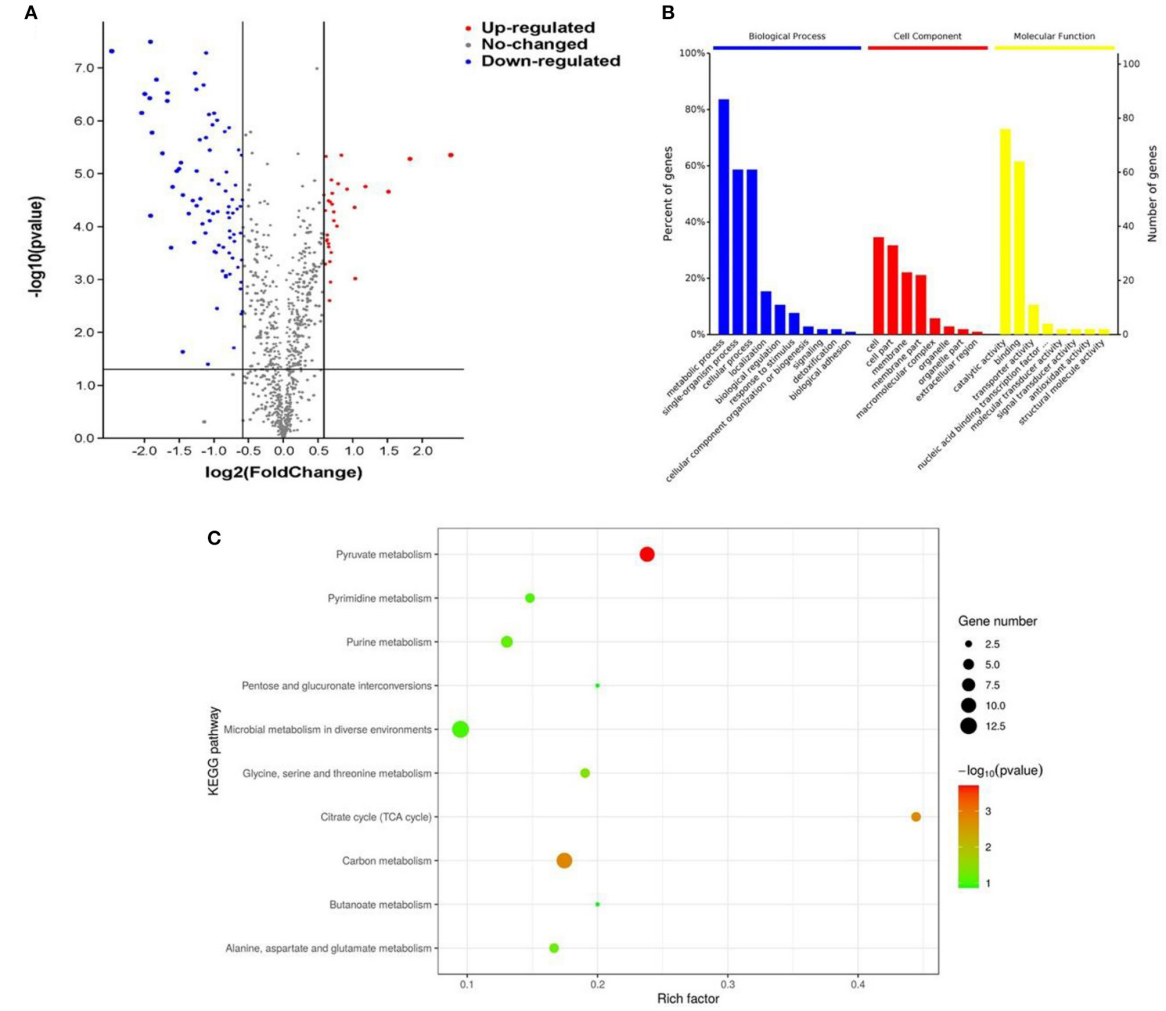
The effects of *L. plantarum* KM1 on the protein level under the stress of H_2_O_2_. Changes in protein levels **(A)**, functional classification of the Gene Ontology (GO) **(B)** and the distribution of differentially expressed proteins in the Kyoto Encyclopedia of Genes and Genomes (KEGG) pathway (top 10) **(C)** in *L. plantarum* KM1 under H_2_O_2_ stress. Red: upregulate differentially expressed proteins; blue: downregulate differentially expressed proteins.

Differentially expressed proteins of *L. plantarum* KM1 were analyzed. The results showed that a total of 112 proteins were differently expressed, of which 31 DEPs such as phosphoglycerate kinase (PGK), homoserine dehydrogenase, and hypothetical protein were upregulated and 81 DEPs were downregulated such as transaldolase (TAL) and pyruvate oxidase (Figure 4A).

The GO function enrichment of DEPs was mainly composed of three functional categories, namely, biological process (BP), cellular component (CC), and molecular function (MF) (Ashburner et al., 2000). DEPs of *L. plantarum* KM1 were mainly related to the metabolic process, single-organism process, cellular process, and detoxication of BP, and 91 DEPs participated in the metabolic process of BP. At same time, the DEPs exerted antioxidant activity, catalytic activity, binding, and transport activity in oxidation-reduction process of MF, and 82 of them participated in the catalytic activity of MF after H_2_O_2_ treatment (Figure 4B).

Kyoto Encyclopedia of Genes and Genomes pathway enrichment analysis was used to further explore the main pathway of *L. plantarum* KM1 after H_2_O_2_ stress (Figure 4C). The most significant pathway was pyruvate metabolism, and the second one was carbon metabolism and citrate cycle (TCA cycle) from the KEGG pathway enrichment of DEPs. Moreover, the expression changes in proteins in these pathways may be related to the antioxidation of the strain. In addition, the KEGG enrichment pathway also showed that some DEPs were enriched in glycine, serine, and threonine metabolism. Among the microbial metabolism in diverse environments pathways, the DEPs quantity of *L. plantarum* KM1 was the largest, and the second one was pyruvate metabolism and carbon metabolism. The results indicated that *L. plantarum* KM1 responded to the environmental stress of H_2_O_2_ by pyruvate metabolism, carbon metabolism, TCA cycle, and microbial metabolism in diverse environments based on KEGG analysis from DEPs.

#### Carbohydrate Transport and Metabolism of *L. plantarum* KM1 Under H_2_O_2_ Stress

Microorganisms use carbon sources as nutrients to carry out life activities. The transportation and metabolism of carbohydrates are vital to the growth and metabolism of the strain. The phosphotransferase system (PTS) system galactitol-specific EIIC component was upregulated by 2.86-fold after H_2_O_2_ stimulation (Table 2). Beta-phosphoglucomutase (β-PGM) was downregulated by 0.64-fold (Table 2), which catalyzed the mutual conversion of β-D-glucose-1-phosphate and β-D-glucose-6-phosphate, while β-D-glucose-1-phosphate played a role in the biosynthesis and degradation of polysaccharides and was the product of the phosphating effect of maltose and trehalose. Meanwhile, maltodextrin-binding protein MdxE and maltose transport system permease protein MalF were downregulated by 0.35- and 0.63-fold (Table 2), respectively, which participated in the transport and metabolism of maltose. Trehalose import ATP-binding protein SugC involved in the transport and metabolism of trehalose was downregulated by 0.59-fold under the stress of H_2_O_2_.

**Table 2:** Differentially expressed proteins (DEPs) of *L. plantarum* KM1 under H_2_O_2_ stress.

**Accession**	**Description**	**Ratio (H_**2**_O_**2**_/control)**	***P*-value (H_**2**_O_**2**_/control)**
**PTS system/ABC transporter/carbohydrate metabolism**			
lclCP055123.1_prot_QKX11280.1_2571	PTS system galactitol-specific EIIC component	2.86	2.19478E-05
lclCP055123.1_prot_QKX11008.1_2299	Maltodextrin-binding protein MdxE	0.35	8.13248E-06
lclCP055123.1_prot_QKX11007.1_2298	Maltose transport system permease protein MalF	0.63	4.65716E-05
lclCP055123.1_prot_QKX11003.1_2294	Trehalose import ATP-binding protein SugC	0.59	0.000120887
lclCP055123.1_prot_QKX11105.1_2396	Beta-phosphoglucomutase	0.64	3.55735E-06
**Glycolysis**			
lclCP055123.1_prot_QKX10546.1_1837	Phosphoglycerate kinase	1.06	0.006403339
lclCP055123.1_prot_QKX10486.1_1777	Xaa-Pro dipeptidyl-peptidase	1.50	2.52263E-05
**Pentose phosphate pathway**			
lclCP055123.1_prot_QKX11288.1_2579	Transaldolase	0.44	2.97974E-05
lclCP055123.1_prot_QKX11679.1_2970	Putative oxidoreductase	0.48	7.7401E-05
**Pyruvate metabolism**			
lclCP055123.1_prot_QKX08955.1_246	Pyruvate oxidase	0.46	2.08125E-06
lclCP055123.1_prot_QKX09342.1_633	Pyruvate dehydrogenase E1 component subunit alpha	0.30	4.12662E-06
lclCP055123.1_prot_QKX09343.1_634	Pyruvate dehydrogenase E1 component subunit beta	0.33	1.77578E-05
**TCA cycle**
lclCP055123.1_prot_QKX11683.1_2974	Succinate-semialdehyde dehydrogenase [NADP(+)] 1	0.45	8.87979E-05
lclCP055123.1_prot_QKX09345.1_636	Dihydrolipoyl dehydrogenase	0.42	4.03037E-05
lclCP055123.1_prot_QKX09344.1_635	Dihydrolipoyllysine-residue acetyltransferase component of pyruvate dehydrogenase complex	0.46	0.000132282
**Oxidation-reduction and antioxidant**			
lclCP055123.1_prot_QKX10677.1_1968	Homoserine dehydrogenase	1.57	0.000242095
lclCP055123.1_prot_QKX11816.1_3107	Anaerobic ribonucleoside-triphosphate reductase	1.60	3.41515E-05
lclCP055123.1_prot_QKX11388.1_2679	Hypothetical protein	5.32	4.44335E-06
lclCP055123.1_prot_QKX11823.1_3114	HTH-type transcriptional regulator GmuR	0.31	4.20921E-07
lclCP055123.1_prot_QKX11281.1_2572	D-arabitol-phosphate dehydrogenase	0.37	0.023332187
lclCP055123.1_prot_QKX09754.1_1045	Acyl carrier protein	0.37	2.5358E-05
lclCP055123.1_prot_QKX11683.1_2974	Succinate-semialdehyde dehydrogenase [NADP(+)] 1	0.45	8.87979E-05
lclCP055123.1_prot_QKX08952.1_243	Thioredoxin-like protein YtpP	0.57	5.45829E-05
lclCP055123.1_prot_QKX10281.1_1572	NAD-dependent malic enzyme	0.67	0.004007364
lclCP055123.1_prot_QKX09023.1_314	NADH peroxidase	0.56	2.12815E-05
lclCP055123.1_prot_QKX08815.1_106	Flavodoxin	0.52	9.75999E-07
lclCP055123.1_prot_QKX10538.1_1829	SsrA-binding protein	0.65	0.000132778

Phosphoglycerate kinase and Xaa-Pro dipeptidyl-peptidase participated in the metabolic pathways of glycolysis. PGK could catalyze the conversion of glycerate-1, 3 P_2_ to glycerate-3 P, which was conducive to produce more ATP in glycolysis pathway. PGK of KM1 was upregulated only 1.06-fold under H_2_O_2_ stress (Table 2). Xaa-Pro dipeptidyl-peptidase catalyzed glyceraldehyde-3-phosphate production and provided NADH and H^+^ for the subsequent conversion of pyruvate to lactic acid. It was upregulated by 1.5-fold after stimulation by H_2_O_2_ (Table 2).

Transaldolase and other DEPs indirectly participated in glycolysis and TCA cycle through pentose phosphate pathway (PPP). TAL was downregulated by 0.44-fold in *L. plantarum* KM1 under H_2_O_2_ stress (Table 2). The putative oxidoreductase was downregulated by 0.48-fold under the stimulation of H_2_O_2_, which eventually formed ribulose 5 phosphate.

Pyruvate metabolism plays a vital role in oxidative stress. Pyruvate oxidase not only responded to H_2_O_2_ stress through the pyruvate metabolic pathway but also participated in the oxidation-reduction process. Pyruvate oxidase was down-regulated by 0.46-fold in *L. plantarum* KM1 under H_2_O_2_ stimulation (Table 2) that induced the conversion of pyruvate to acetyl phosphate was inhibited, and the H_2_O_2_ production was reduced in cells. The pyruvate dehydrogenase complex is composed of pyruvate dehydrogenase (E1), dihydrolipoyl acetyltransferase (E2), and dihydrolipoyl dehydrogenase (E3). After H_2_O_2_ stress, pyruvate dehydrogenase E1 component subunit alpha and pyruvate dehydrogenase E1 component subunit beta were, respectively, downregulated by 0.30- and 0.33-fold, which reduced the conversion of pyruvate to acetyl-CoA.

The TCA cycle is the metabolic hub of the three major nutrients and energy. Dihydrolipoyl dehydrogenase (pyruvate dehydrogenase complex E3 component) and dihydrolipoyllysine-residue acetyltransferase component of pyruvate dehydrogenase complex (pyruvate dehydrogenase complex E2 component) were, respectively, downregulated by 0.42- and 0.46-fold under H_2_O_2_ stress. They catalyzed the conversion of pyruvate to acetyl-CoA, reducing the acetyl-CoA production. Succinate-semialdehyde dehydrogenase [NADP(+)] 1 was downregulated by 0.45-fold under H_2_O_2_ stress, which reduced the conversion of succinic semialdehyde to succinic acid. The results indicated that the TCA cycle of *L. plantarum* KM1 was inhibited by H_2_O_2_.

#### The Oxidation-Reduction Process and Antioxidant Effect of *L. plantarum* KM1 Under H_2_O_2_ Stress

The cell should maintain a certain oxidation-reduction homeostasis to resist oxidative stress (Shimizu and Matsuoka, 2019). Homoserine dehydrogenase, anaerobic ribonucleoside-triphosphate reductase, and hypothetical protein all participated in the oxidation-reduction process, which were upregulated by 1.57-, 1.60-, and 5.32-fold under H_2_O_2_ stress (Table 2), respectively. In addition, some DEPs also participated in the oxidation-reduction process and were downregulated by 0.31–0.67 times under H_2_O_2_ stress, including HTH-type transcriptional regulator GmuR, D-arabitol-phosphate dehydrogenase, acyl carrier protein, succinate-semialdehyde dehydrogenase [NADP (+)] 1, thioredoxin-like protein YtpP, NAD-dependent malic enzyme, flavodoxin, SsrA-binding protein, and NADH peroxidase (Table 2). SsrA-binding protein in KM1 slightly decreased under 5 mM H_2_O_2_ stimulation. In the process of maintaining redox balance, homoserine dehydrogenase, anaerobic ribonucleoside-triphosphate reductase, and hypothetical protein in *L. plantarum* KM1 played a major role.

#### Amino Acid Biosynthesis of *L. plantarum* KM1 Under H_2_O_2_ Stress

**Figure 5:**
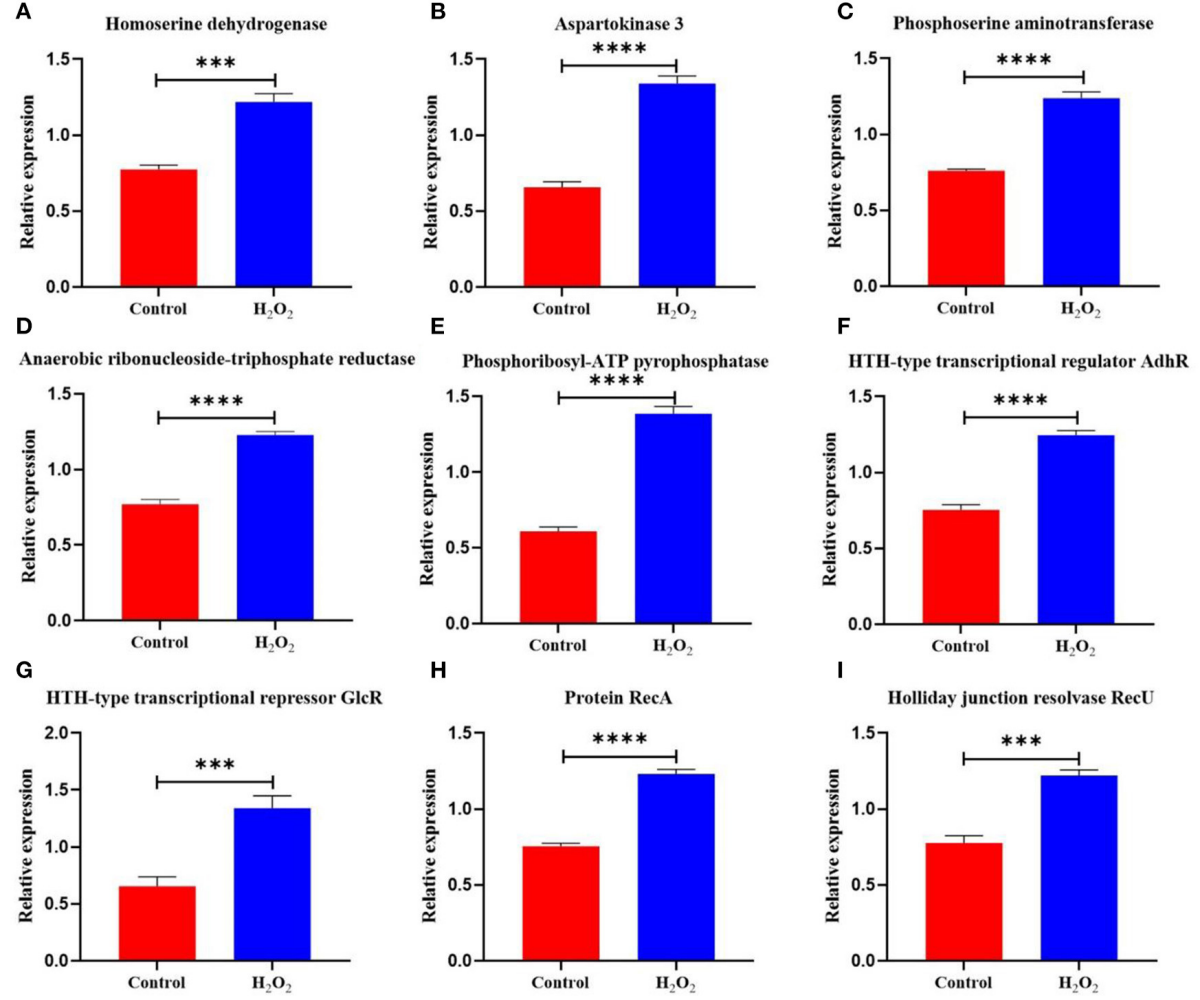
The differentially expressed proteins in the amino acid biosynthesis and DNA repair system of *L. plantarum* KM1 under H_2_O_2_ stress. Red: control group; blue: H_2_O_2_ group. ***p < 0.001; ****p < 0.0001. Homoserine dehydrogenase **(A)**, aspartokinase 3 **(B)**, phosphoserine aminotransferase **(C)**, anaerobic ribonucleoside-triphosphate reductase **(D)**, phosphoribosyl-ATP pyrophosphatase **(E)**, HTH-type transcriptional regulator AdhR **(F)**, HTH-type transcriptional regulator GlcR **(G)**, protein RecA **(H)**, and holliday junction resolvase RecU **(I)**.

Under H_2_O_2_ stress, homoserine dehydrogenase and aspartokinase 3 increased by 1.57- and 2.04-fold (Figures 5A,B), respectively. Phosphoserine aminotransferase catalyzed the production of D-aspartate to resist H_2_O_2_, and the enzyme was upregulated by 1.63-fold in *L. plantarum* KM1 at 5 mM H_2_O_2_ (Figure 5C). Additionally, the TCA cycle was weakened when the pyruvate oxidase and pyruvate dehydrogenase E1 components were downregulated. To improve the adaptability to H_2_O_2_, *L. plantarum* KM1 upregulated proteins related to amino acid synthesis.

#### DNA Repair of *L. plantarum* KM1 Under H_2_O_2_ Stress

Oxidative stress causes DNA damage (Preiser, 2012). Anaerobic ribonucleoside-triphosphate reductase [EC:1.1.98.6] in *L. plantarum* KM1 was upregulated by 1.60-fold under H_2_O_2_ stress (Figure 5D) and catalyzed the production of dGTP for DNA synthesis and repair. Phosphoribosyl-ATP pyrophosphatase for DNA repair and recombination in response to H_2_O_2_-induced oxidative stress were upregulated by 2.27-fold (Figure 5E). Under H_2_O_2_ stress, HTH-type transcriptional regulator AdhR and HTH-type transcriptional repressor GlcR were upregulated by 1.66- and 2.05-fold, respectively (Figures 5F,G). In addition, protein RecA was upregulated by 1.63-fold, which performed homologous recombination DNA repair (Figure 5H). The Holliday junction resolvase RecU of *L. plantarum* KM1 increased by 1.57-fold under H_2_O_2_ stress, which enabled RecA to catalyze DNA strand exchange (Figure 5I). Therefore, *L. plantarum* KM1 could alleviate the DNA damage caused by H_2_O_2_ through the DNA repair system.

### PPI Networks of DEPs in *L. plantarum* KM1 Under H_2_O_2_ Stress

**Figure 6:**
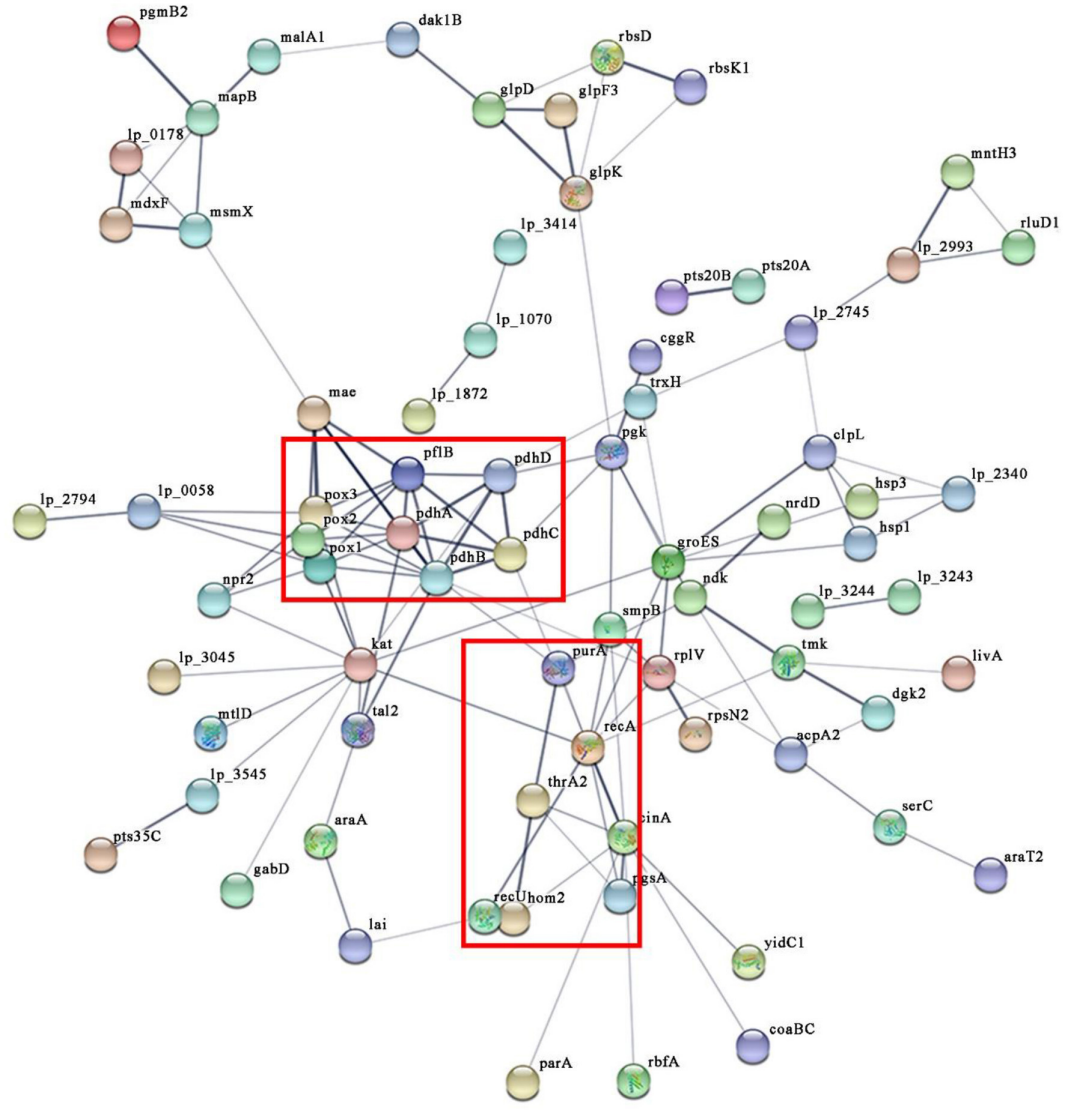
The protein-protein interaction networks of differentially expressed proteins in *L. plantarum* KM1 under H_2_O_2_ stress. Different circles represent different proteins, and there is a line in the middle indicating that there may be an interaction relationship. pox1, pyruvate oxidase; pox2, hypothetical protein; pox3, pyruvate oxidase; pdhA, pyruvate dehydrogenase E1 component subunit alpha; pdhB, pyruvate dehydrogenase E1 component subunit beta; pdhC, dihydrolipoyllysine-residue acetyltransferase component of pyruvate dehydrogenase complex; pdhD, dihydrolipoyl dehydrogenase; pflB, formate acetyltransferase; mae, NAD-dependent malic enzyme; gabD, succinate-semialdehyde dehydrogenase [NADP(+)] 1; npr2, NADH peroxidase; kat, SsrA-binding protein; hom2, homoserine dehydrogenase; thrA2, aspartokinase 3; purA, adenylosuccinate synthetase; recA, protein RecA; recU, holliday junction resolvase RecU (abbreviation for important protein).

To observe the interaction of the DEPs of *L. plantarum* KM1 under H_2_O_2_ stress, the PPI network was analyzed (Figure 6). The most obvious PPI network of *L. plantarum* KM1 was pyruvate metabolism and TCA cycle (pox, pdhA, pdhB, phhC, pdhD). Among them, pyruvate oxidase (pox) played an important role in the detoxification of H_2_O_2_. NADH peroxidase (npr2), NAD-dependent malic enzyme (mae), and SsrA-binding protein (kat) interacted more with other proteins and played an antioxidant role. In addition, the upregulated DEPs related to DNA repair (recA, recU) and amino acid biosynthesis (hom2, thrA2) formed an interaction network that could be involved in reducing the cell damage caused by oxidative stress to *L. plantarum* KM1, which was consistent with all the above descriptions.

### Transcription Level Validation in *L. plantarum* KM1 Under H_2_O_2_ Stress

**Figure 7:**
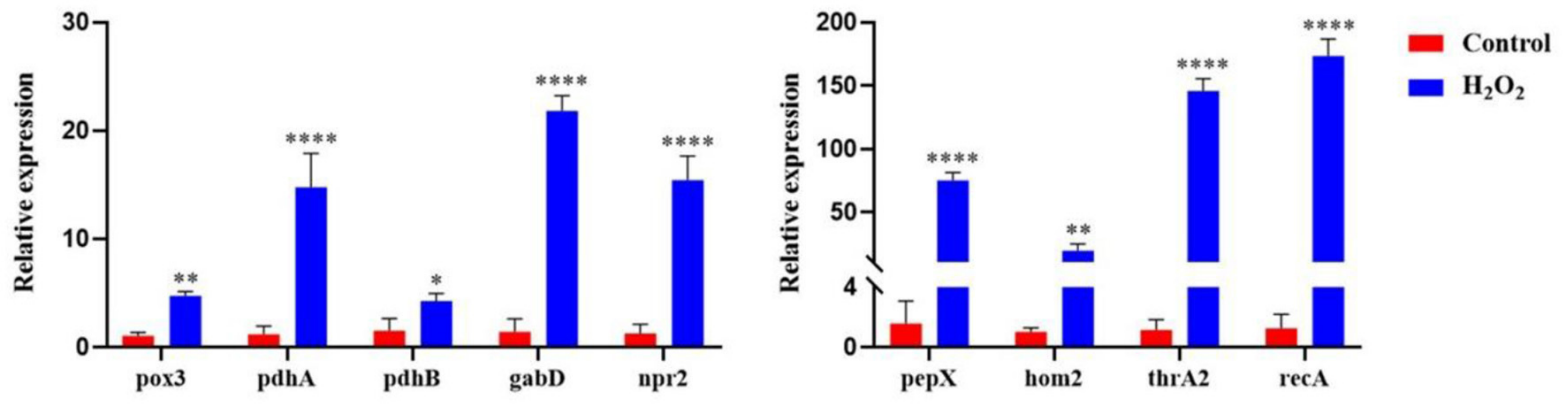
The relative expression of genes encoding DEPs in *L. plantarum* KM1 under H_2_O_2_ stress Red: control group; blue: H_2_O_2_ group. *p < 0.05; **p < 0.01; and ****p < 0.0001.

To verify the reliability of the protein level changes of *L. plantarum* KM1 under H_2_O_2_ stress, the relative expression changes of the genes were measured at the transcriptional level (Figure 7). The results showed that under H_2_O_2_ stress, the relative expression levels of 9 genes that played key roles in each pathway were verified to be significantly upregulated. Among them, the gene pepX encoding Xaa-Pro dipeptidyl-peptidase was involved in the glycolytic metabolic pathway and was up-regulated 74.89-fold (*p* < 0.0001); the genes pox3 (pyruvate oxidase) and pdhA (pyruvate dehydrogenase E1 component subunit alpha) were involved in pyruvate metabolism, which were upregulated by 4.75- and 14.76-fold after H_2_O_2_ treatment, respectively. The gene pdhB encoding pyruvate dehydrogenase E1 component subunit beta and the gene gabD encoding succinate-semialdehyde dehydrogenase [NADP(+)] 1 were involved in the TCA cycle, and the gene expression levels were upregulated by 4.27- and 21.80-fold, respectively. The gene npr2 encoding NADH peroxidase had antioxidant effects, and under oxidative stress, it was upregulated by 7.64-fold, respectively. The hom2 (homoserine dehydrogenase) and the thrA2 (aspartokinase 3) were involved in the biosynthesis of amino acids. After H_2_O_2_ treatment, the expression levels were upregulated by 19.47- and 145.81-fold, respectively. While the gene expression of recA (RecA protein) was upregulated by 173.44-fold, the corresponding protein was related to DNA repair.

## Discussion

*L. plantarum* KM1 had antioxidant capacity and could resist oxidative stress in the environment. Studies had shown that *L. plantarum* KM1 tolerated up to 5 mM H_2_O_2_, which was significantly higher than the H_2_O_2_ inhibiting concentration (2.5 mM H_2_O_2_) of *L. plantarum* MA2 (Tang et al., 2017). In addition, *L. plantarum* needed to have probiotic properties that could tolerate the human gastrointestinal environment before it could exert important antioxidant functions. *L. plantarum* KM1 grew normally under the condition of pH 2.5, and the survival rate of culturing for 2 h was 96.75%. However, the number of viable cells of *L. plantarum* L3C1E8 decreased from 8.84 ± 0.23 to 2.25 ± 0.14 log CFU/ml at pH 2.5 for 2 h, and its survival rate was only 25.45% (Ribeiro et al., 2018), which was evidently lower than that of *L. plantarum* KM1. The survival rate of *L. plantarum* KM1 cultured at 0.2% bile salt concentration for 2 h was 80.26%, which was significantly higher than *L. plantarum* RW1 at the same concentration for 3 h (75%) (Raheem et al., 2021). Antibiotics including kanamycin sulfate, streptomycin sulfate, tetracycline HCl, and ampicillin trihydrate inhibited protein synthesis (Oh and Jung, 2015). Therefore, co-administration with these antibiotics inhibited the growth of *L. plantarum* KM1 and reduced protein synthesis. Moreover, the MIC of *L. plantarum* KM1 against the five antibiotics all met the breakpoints provided by EFSA, where kanamycin sulfate was higher than the breakpoint of EFSA (FEEDAP, 2012). The MIC of *L. plantarum* KM1 on kanamycin sulfate and streptomycin sulfate was consistent with the breakpoints provided by Danielsen and Wind (2003). It was determined by *in vitro* tests that *L. plantarum* KM1 had certain probiotic properties and met the screening standards for probiotics.

Then, the antioxidant capacity of *L. plantarum* KM1 was evaluated, and the results showed the antioxidant capacity from strong to weak was fermentation supernatant, cell-free extract, and intact cells. The reducing capacity of *L. plantarum* KM1 cell-free extracts (310.67 ± 12.019 μmol/L) and intact cells (271.778 ± 4.006 μmol/L) were both higher than *L. plantarum* MA2 (88.38 ± 3.01 μmol/L, 154.33 ± 4.79 μmol/L) (Tang et al., 2017). The ∙OH scavenging rate of the KM1 fermentation supernatant was significantly higher than *L. plantarum* CD101 (18.6%) (Luan et al., 2021). The ∙OH scavenging rate of the cell-free extract (66.963 ± 1.033%) was also higher than *L. plantarum* CD101 (11.6%), which could effectively alleviate oxidative damage caused by ROS (Luan et al., 2021). In addition, the DPPH free radical scavenging rate of *L. plantarum* KM1 fermentation supernatant was higher than *L. plantarum* C88 (53.05%) (Li et al., 2012). The DPPH scavenging of the KM1 cell-free extract in the control was lower than *L. plantarum* MA2 (40.42 ± 2.19%) but higher than MA2 (Tang et al., 2017) when it was treated with H_2_O_2_ (46.240 ± 1.051%). Although H_2_O_2_ treatment sometimes decreased the antioxidant capacity of *L. plantarum* KM1, overall, the decrease was less compared with the control group.

Since *L. plantarum* KM1 has good antioxidant capacity *in vitro*, the role of DEPs of *L. plantarum* in oxidative stress was determined by proteomics. GO functional enrichment revealed functional annotation of DEPs in *L. plantarum*. In particular, the detoxification shown by NADH peroxidase in GO functional enrichment helped strains cope with oxidative stress. In addition, some antioxidant enzymes exerted their antioxidant activity in MF when the strain responded to H_2_O_2_ stress. Among the antioxidant enzymes, NADH peroxidase participated in the oxidation-reduction process, which converted H_2_O_2_ into H_2_O (Gordon et al., 1953; Sakamoto and Komagata, 1996). The activity of NADH peroxidase regulated degradation rate of H_2_O_2_, and was directly proportional to the oxygen availability (Kang et al., 2013). The corresponding gene for the SsrA binding protein is kat, a gene for catalase that can reduce H_2_O_2_ to water and oxidize it to molecular oxygen (Galasso et al., 2021). The downregulation of SsrA binding protein, NADH peroxidase, and other antioxidant enzymes in the strain might be due to the fact that H_2_O_2_ was consumed when the strain was in the lag phase, and the expression of antioxidant enzymes in the strain decreased. Moreover, the GO function enrichment of *L. plantarum* CAUH2 at the transcription level also exhibited antioxidant activity and transport activity of MF, which was consistent with the findings of *L. plantarum* KM1 (Zhai et al., 2020).

The DEPs of *L. plantarum* KM1 responded to H_2_O_2_ stress by exerting its important roles in seven metabolic pathways: PTS system, glycolysis, PPP, pyruvate metabolism, TCA cycle, amino acid biosynthesis, and DNA repair system. *L. plantarum* needs to absorb nutrients from the MRS medium to survive after being stimulated by H_2_O_2_, and the strain takes glucose through the PTS. The PTS is an important condition for the transportation and application of carbon sources, including galactitol, mannitol, fructose, sorbose, and cellobiose (Zhang et al., 2010; Joyet et al., 2013; He et al., 2018). The PTS system galactitol-specific EIIC component was involved in the biosynthesis of peptidoglycan and played an important role in the transportation of carbon sources (Lu et al., 2020). Therefore, the upregulation of PTS-related proteins increased the carbon sources utilization by improving the transportation and metabolism of carbohydrates to resist the stimulation of H_2_O_2_. In addition, the downregulation of maltose transport system permease protein MalF in *L. plantarum* KM1 under the stimulation of H_2_O_2_ was opposite to the RNA change trend of the corresponding gene in *L. plantarum* CAUH2 (Zhai et al., 2020). This might be due to a certain difference in the transcription level and the protein expression level.

Glucose ingested by PTS undergoes glycolysis to pyruvate and ATP. PGK, as a key enzyme in the glycolytic pathway, directly phosphorylated the ADP to generate ATP (Merli et al., 2002). The change trend of PGK was consistent with the corresponding gene in *L. plantarum* CAUH2 (Zhai et al., 2020). The upregulation of PGK and Xaa-Pro dipeptidyl-peptidase showed *L. plantarum* responded to H_2_O_2_ stress by enhancing glycolysis to produce more energy.

At the same time, glucose could also enter the PPP without glycolysis to produce NADPH for biosynthesis. PPP was divided into the non-oxidizing part and oxidizing part (Chesworth et al., 2008). According to related studies, the TAL involved in the PTS system was the rate-limiting enzyme in the non-oxidative part of the PPP (Lachaise et al., 2001). The decrease of TAL activity led to the increase of the NADPH level, which inhibited the production of ROS and cell damage (Samland and Sprenger, 2009). Therefore, the downregulation of TAL expression increased *L. plantarum* KM1 resistance to oxidative stress induced by H_2_O_2_. Moreover, the oxidation part of the PPP is the process of converting glucose-6-phosphate into ribulose-5-phosphate, which is also accompanied by NADPH production (Tang, 2019). The putative oxidoreductase acted on NAD^+^ or NADP^+^ acceptor, and its downregulation reduced the use of NAD^+^ or NADP^+^ that increased NADPH production of PPP to reduce cell damage.

The pyruvate produced by glycolysis is oxidatively decarboxylated to acetyl-CoA under the catalysis of the pyruvate dehydrogenase complex and then enters the TCA cycle through acetyl-CoA. Pyruvate metabolism, carbon metabolism and TCA cycle were affected by the oxidation-reduction state (Shetty and Varshney, 2021). Pyruvate metabolism inhibits the production of H_2_O_2_ and pyruvate as the final product of glycolysis participated in the TCA cycle (Crestanello et al., 1998; Liu et al., 2009). FAD and TPP were used by pyruvate oxidase as cofactors to catalyze the reaction of pyruvate and phosphate to form H_2_O_2_ (Lorquet et al., 2004). The downregulation of it greatly reduced endogenous H_2_O_2_, which was beneficial for the strain to cope with H_2_O_2_ stress. The pyruvate dehydrogenase complex mainly converted pyruvate to acetyl-CoA (Grabsztunowicz et al., 2020). The downregulation of DEPs associated with the pyruvate dehydrogenase complex reduced the NADH and ROS production by mitochondrial respiration and maintained cell homeostasis (Anand et al., 2021). However, the TCA cycle captured most of the energy released by glucose oxidation, while downregulation of DEPs on the TCA cycle pathway weakened the TCA cycle and reduced energy production (Fernie et al., 2004). In addition, bacterial growth is inseparable from energy. Furthermore, H_2_O_2_ treatment downregulated DEPs associated with the TCA cycle. Therefore, the growth of the strain slowed down significantly with the increase in H_2_O_2_ concentration (Figure 1D), which might be due to insufficient energy.

Attenuation of the TCA cycle shifted phosphoenolpyruvate toward amino acid biosynthesis. Studies showed that glycine, serine, and threonine metabolism were closely related to the antioxidant activity (Alhasawi et al., 2015). *L. plantarum* synthesized amino acids including methionine and D-aspartate to resist oxidative stress (Zhai et al., 2020; Kajitani et al., 2021). Homoserine dehydrogenase and aspartokinase 3 were both involved in the synthesis of L-homoserine, which was the precursor of methionine, and L-homoserine had certain stress resistance (Wei et al., 2019; Liu et al., 2020; Tang et al., 2021). Thus, their marked upregulation increased the ability of the strains to resist oxidative stress. In addition, the weakening of TCA cycle increased the conversion of phosphoenolpyruvate to aspartate and finally improved the homoserine synthesis, which was consistent with previous works (Zhang et al., 2021).

Finally, *L. plantarum* KM1 alleviated DNA damage through the DNA repair system. HTH-type transcriptional regulator AdhR and HTH-type transcriptional repressor GlcR belonged to the MerR family. The N-terminal DNA binding region of this family was highly conserved, which played an important role in DNA binding (Brown et al., 2003; Hayashi et al., 2013; Romero-Rodriguez et al., 2015). Particularly, both the protein expression level and the PPI network showed that the protein RecA played a vital role in the DNA repair system, infusing the strain with powerful antioxidant capacity. The expression trend of the gene recA of *L. plantarum* KM1 at the transcriptional level was consistent with that of its corresponding DEPs (protein RecA). In addition, the expression of gene recA of *L. plantarum* KM1 in this study was consistent with the corresponding gene transcription level of *L. plantarum* CAUH2 (Zhai et al., 2020).

## Conclusion

*L. plantarum* KM1 with certain intestinal viability was derived from natural fermented food, which exhibited good antioxidant activity *in vitro*. The proteomics analysis indicated that DEPs participated in various metabolic pathways including pyruvate metabolism, carbon metabolism, TCA cycle, amino acid metabolism, and microbial metabolism in diverse environments in response to oxidative stress caused by H_2_O_2_ in *L. plantarum* KM1. In addition, glycolysis and DNA repair also played an important role in dealing with H_2_O_2_ stress.

## Data Availability Statement

The original contributions presented in the study are included in the article/Supplementary Material, further inquiries can be directed to the corresponding authors. The data presented in the study are deposited in the ProteomeXchange repository, accession number PXD032910. The names of the repository/repositories and accession number(s) can be found at: https://www.ebi.ac.uk/pride/archive, PXD032910.

## Author Contributions

YT: conceptualization, investigation, formal analysis, writing—original draft, and preparation. YuW, JZ, and BN: writing—review and editing. NZ, MX, XX, YZ, and YF: technical support. XL, YuhW, and JL: conceptualization, resources, supervision, funding acquisition, project administration, and writing—review and editing. All authors contributed to the article and approved the submitted version.

## Funding

This research was a part of the project funded by the Jilin Province Science and Technology Development Plan Project (20200403163SF).

## Conflict of Interest

The authors declare that the research was conducted in the absence of any commercial or financial relationships that could be construed as a potential conflict of interest.

## Publisher's Note

All claims expressed in this article are solely those of the authors and do not necessarily represent those of their affiliated organizations, or those of the publisher, the editors and the reviewers. Any product that may be evaluated in this article, or claim that may be made by its manufacturer, is not guaranteed or endorsed by the publisher.

## Abbreviations

ABTS: 3-ethylbenzothiazoline-6-sulfonic acid
ACN: acetonitrile
DEPC: diethyl pyrocarbonate
DPPH, 1: 1-diphenyl-2-picrylhydrazyl radical
DTT: dithiothreitol
GRAS: generally recognized as safe
SDT, 4% sodium lauryl sulfate (SDS), 100mM Tris/HCl pH 7.6: 0.1M DTT
TMT: tandem mass tag.
