# Supplementary material for: Antioxidant Mechanism of Lactiplantibacillus plantarum KM1 Under H2O2 Stress by Proteomics Analysis
Source: Front Microbiol. 2022 Jun 27;13:897387. doi: 10.3389/fmicb.2022.897387 (PMC9271951; doi:10.3389/fmicb.2022.897387)
Supplement: Supplementary file 1 [file Data_Sheet_1.docx]

**Table S1 PCR amplification conditions**

| Step | Cycle number | Temperature (°C) | Time (s) |
| --- | --- | --- | --- |
| 1 | 1 | 95 | 30 |
| 2 | 40 | 95 | 10 |
| 3 | 1 | 56 | 30 |
| 4 | 1 | 72 | 30 |

**Table S2 PCR primer design**

| Gene | Accession | Sequence (5’ -3’) | Bases (bp) |
| --- | --- | --- | --- |
| pox3 | CCC79767.1 | ATCTGCTTTAGCCGCTTGGT  AGTTTGGTCGGCACCATCAT | 20 |
| pdhA | CCC79382.1 | ACCGGTATGTCAGCGTTTCA  TTGCTGCCGGTATTCCAAGT | 20 |
| pdhB | CCC79381.1 | TTGGCCGGAAACCTTCCAAT  CCTGCAAGCCGAATTTGGTG | 20 |
| gabD | CCC80136.1 | TAGGTGGTTCGGACGCTTTC  ACACATCCGCAGCGACAATA | 20 |
| npr2 | CCC79703.1 | CACGTCACGGTTATCGACCT  TTGGCCAACTGCAGCATAGA | 20 |
| pepX | CCC78308.1 | TGTCATAGCCGTGCCAAGTT  TCGCTCGATTTCACCGACAT | 20 |
| hom2 | CCC78057.1 | AACGGGTCATGGGAATCGTC  TTTCGGCAAACCCCAAGTCT | 20 |
| thrA2 | CCC79507.1 | ACATCAGTTGACGCCCGAAT  CATGTTTTCGCCAACGAGCA | 20 |
| recA | CCC79501.1 | ACCTTTTGGCGAATCTCCGT  ATGGTGACGATCGCATTGGT | 20 |
| 16sRNA | GCA_000203855.3 | ACCTTCCTCCGGTTTGTCAC  CAGCTCGTGTCGTGAGATGT | 20 |
